# Unreported births and deaths, a severe obstacle for improved neonatal survival in low-income countries; a population based study

**DOI:** 10.1186/1472-698X-8-4

**Published:** 2008-03-28

**Authors:** Mats Målqvist, Leif Eriksson, Nguyen Thu Nga, Linn Irene Fagerland, Dinh Phuong Hoa, Lars Wallin, Uwe Ewald, Lars-Åke Persson

**Affiliations:** 1 International Maternal and Child Health (IMCH), Department of Women's and Children's Health, Uppsala University, SE-751 85 Uppsala, Sweden; 2 Vietnam Sweden Uong Bi General Hospital, Quang Ninh, Vietnam; 3 Department of Reproductive Health, Ministry of Health, Hanoi, Vietnam; 4 Department of Neurobiology, Care Sciences and Society, Division of Nursing, Karolinska Institutet and Clinical Research Utilization (CRU), Karolinska University Hospital, SE- 17176 Stockholm, Sweden; 5 Neonatology, Department of Women's and Children's Health, Uppsala University, SE-751 85 Uppsala, Sweden

## Abstract

**Background:**

In order to improve child survival there is a need to target neonatal mortality. In this pursuit, valid local and national statistics on child health are essential. We analyze to what extent births and neonatal deaths are unreported in a low-income country and discuss the consequences at local and international levels for efforts to save newborn lives.

**Methods:**

Information on all births and neonatal deaths in Quang Ninh province in Northern Vietnam in 2005 was ascertained by systematic inventory through group interviews with key informants, questionnaires and examination of health facility records. Health care staff at 187 Community Health Centers (CHC) and 18 hospitals, in addition to 1372 Village Health Workers (VHW), were included in the study. Results were compared with the official reports of the Provincial Health Bureau.

**Results:**

The neonatal mortality rate (NMR) was 16/1000 (284 neonatal deaths/17 519 births), as compared to the official rate of 4.2/1000. The NMR varied between 44/1000 and 10/1000 in the different districts of the province. The under-reporting was mainly attributable to a dysfunctional reporting system and the fact that families, not the health system, were made responsible to register births and deaths. This under-reporting has severe consequences at local, national and international levels. At a local level, it results in a lack of awareness of the magnitude and differentials in NMR, leading to an indifference towards the problem. At a national and international level the perceived low mortality rate is manifested in a lack of investments in perinatal health programs.

**Conclusion:**

This example of a faulty health information system is reportedly not unique in low and middle income countries where needs for neonatal health reforms are greatest. Improving reporting systems on births and neonatal deaths is a matter of human rights and a prerequisite for reducing neonatal mortality in order to reach the fourth millennium goal.

## Background

It is increasingly acknowledged that the neonatal period has been neglected in the pursuit of improved child survival [1] and that deaths in the neonatal period (the first 28 days after delivery) today constitute an increasing proportion of the overall under-5 mortality[2]. This neglect is not only a matter of withheld interventions, but also very much a question of invisibility of the problem[3]. Thousands of newborns are never registered as being born. Within this group, the rate of neonatal deaths is higher than in the rest of the population as many unregistered neonatal deaths die before the registration of birth[4]. This invisibility of neonatal deaths is a severe obstacle in the pursuit of improved neonatal survival since it precludes adequate planning and measures by local and global actors. In order to reach those in greatest need there has been a call for universal access to skilled birth attendance[5] and community-based interventions[6], which have been shown to be cost-effective and well-suited for scaling up[7,8]. These interventions can not rely on estimates from national or regional levels, but must be based in the local context. Therefore, there is a need for valid health statistics[9,10]and data reporting on child births and deaths in order to have a sound basis for design and monitoring of interventions for improved child survival[8].

Vietnam has reported a low neonatal mortality rate (NMR) in the official statistics for the past couple of years compared to other countries in equivalent socioeconomic situations. According to the Demographic and Health Survey of 2002, Vietnam has a NMR of 12.2/1000 [11], a figure used for WHO official statistics as well [12]. If this NMR is correct it will pose questions and hopes about the causes to the relative success in the field of child health of a country in the lower half of the income scale. However, studies indicate that there might be considerable under-reporting of neonatal deaths in the official registers[13] and difficulties in obtaining correct information on neonatal deaths even in population-based studies. Considering the importance of the official figures at all levels for policy formation, planning and allocation of funds, it is evident that accurate information is needed on the health situation of newborns in low- and middle-income countries. The aim of the present study is to analyse to what extent births and neonatal deaths go unreported in the official statistics in a province in Vietnam, and to discuss the consequences at local and national levels for future efforts to save newborn lives.

## Methods

### Setting

The study area was Quang Ninh province, one out of 64 provinces in Vietnam. Quang Ninh is situated along the Vietnamese coastline bordering China in the north, with a population reaching just above 1 million, 80% of which live in rural and mountainous areas. Ha Long City, the major city in the province, is one of Vietnam's major tourist centres, tourism being the driving force of the present economic boom in Quang Ninh. Within this population, 11% belong to ethnic minorities and 10–15% are considered poor[14]. The province is divided into 14 districts and 184 communes. This division is also the base for the health system, with each district containing one district hospital (DH) and each commune containing at least one community health centre (CHC). At the provincial level there is one provincial hospital (PH) situated in Ha Long city and one regional hospital in Uong Bi Town (UBGH).

The reporting of health statistics follows the structure of the health system, with the CHC being the lowest level at which data is recorded in registers. Births and deaths that occur in a commune should all be registered at the CHC, using information from monthly verbal reports from the Village Health Workers (VHW) of that commune. The information is then sent upwards in the system as aggregates, first to the District Health Bureau (DHB) where the figures for the whole district are compiled and then on to the Provincial Health Bureau (PHB). In our study, the official statistics were collected from the data sent to the Ministry of Health from the PHB.

Aside from the health system there are alternate reporting systems through governmental administrative bodies. When a child is born, the family receives a birth certificate from the health institution where the delivery took place or from the CHC if it was a home delivery. With this certificate, the family should register at the Community's People's Committee, providing an alternate source of official statistics on births. The Vietnamese Government has issued a decree which requires parents to register their child within 30 days of birth (60 days in remote areas)[15]. However, even if the child is registered within the prescribed time period it is not necessarily within the neonatal time period, implying that neonatal deaths take place before children need to be registered. When there is a death, there is no death certificate, and the family seldom registers the event at the People's Committee. At the national level, the General Statistics Office (GSO) in Hanoi annually performs a survey, gathering data on infant mortality rate (IMR) and under-5 mortality rate (U5MR) for the whole country. That survey uses a multi-level cluster sampling technique, covering 2% of the population. NMR is not an indicator in that survey.

### Study design

Different sources of information were used to collect data on neonatal mortality in Quang Ninh province for the time period between January and December 2005: health registers at hospitals and CHCs, recollections of reproductive health events by health staff, and group interviews with VHWs. The results were then compared to the official statistics obtained from the Provincial Health Bureau (PHB). Fifteen full-time employed data collectors gathered data from April to June 2006.

#### Checking registers and recollection of health staff

At the regional, provincial and district hospitals, registers at the obstetric and pediatric departments were checked in order to find notations about neonatal deaths. The staff was also asked to recollect cases of neonatal death during 2005 that had not been noted in the books. Before collecting data, a meeting in each district with all the midwives working at CHCs was arranged. At these meetings, information about the study was provided and the midwives got a report form to bring back to the CHC, including data on neonatal deaths. These forms were later cross-checked with the registers of the CHCs.

#### Group interviews with Village Health Workers (VHW)

Every commune is divided into a number of villages and each village has an assigned health worker, mainly responsible for health information and reporting. All in all there were 1500 VHWs in Quang Ninh province. Group interviews, with three to six VHWs at a time, were performed by the data collectors. Verbal consent of participation was obtained before the group interviews took place. The VHWs were asked to recollect any neonatal deaths that happened during 2005 and to give details about these cases. This information was later crosschecked with registers and the recollection of CHC staff.

#### Data analysis

For every neonatal death identified, a separate report form was filled in. The forms were compared in order to find over-lapping information sources and avoid duplicates. Every case of mortality was evaluated according to the WHO definition of neonatal death in order to avoid misclassifications. This process was supervised by the authors.

### Ethical approval

This study was approved by the Ministry of Health in Vietnam, the Provincial Health Bureau in Quang Ninh and the Research Ethics Committee at Uppsala University, Sweden.

## Results

Information was excerpted from registers at all relevant health facilities in Quang Ninh province (n = 205). Questionnaires were received from all midwifes at CHCs (n = 187) and 427 group interviews were conducted with Village Health Workers (VHW). Out of the 1500 villages in the province, 109 were not represented at group interviews (7.3%).

### Birth registration

According to the official statistics gathered from the Provincial Health Bureau there were 16 551 live births in Quang Ninh province during 2005, whereof 854 were born at home. By collecting data from the registers at the different health facilities and adding the information given by the VHWs at group interviews on home deliveries and births taking place outside the province, 17 519 live births in 2005 were found, 1461 of which had occurred outside health facilities (Table 1).

**Table 1:** Births and Neonatal deaths in official statistics and in the present study in Quang Ninh province, Vietnam, 2005

	**Births**	**whereof at home**	**Neonatal deaths**	**NMR**	**95% CI**
**Official statistics from Provincial Health Bureau**	16 551	854	70	4.2	-
**Present study**	17 519	1461	284	16	14–18

### Neonatal deaths

**Figure 1:**
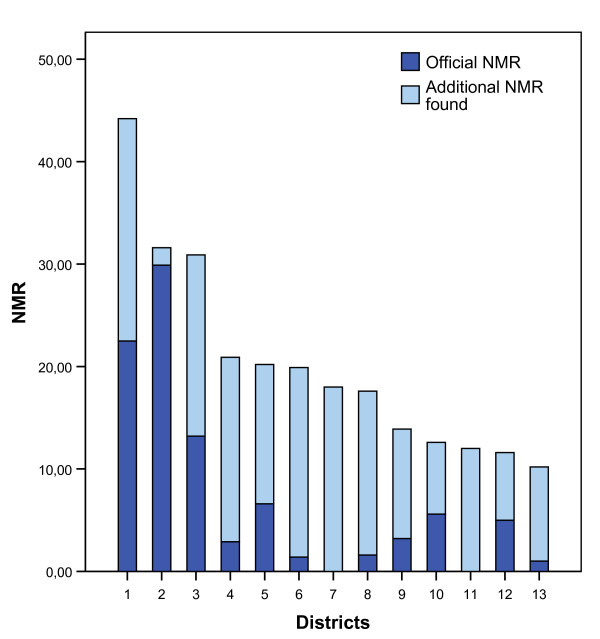
**NMR stratified according to districts in Quang Ninh province revealing the extent of invisible neonatal mortality.** Due to small size, two districts were merged in the analysis, resulting in 13 sub-entities.

A total number of 284 individual neonatal deaths were found through the different methods used, giving a NMR of 16/1000 (284/17 519), using the number of live births and deaths found in our study. The early neonatal deaths (day 0–6) numbered 229/284 (81%) and 131/284 (46%) died within the first 24 hours, 217 (76%) of the deaths occurred at a health facility. When checking registers at health facilities, notations of 213 neonatal deaths were found, whereas the VHWs and CHC staff knew 225 out of the 284 neonatal death cases. Only two cases were found exclusively at the CHC level, as compared to 57 and 54 cases respectively that were found only in the hospital records and when interviewing the VHWs (Table 2). The official statistics from the PHB reported 70 neonatal deaths during 2005, giving an NMR of 4.2/1000 (70/16 551) for the whole province according to the official records. Due to limitations in the official reports, there was no possibility to identify the individual cases of neonatal death for comparison. However, the distribution of NMR at a district level reveals that only one out of 14 districts reported more than 50% of the neonatal deaths. The accuracy of the official statistics vis-à-vis our results varied considerably between the different districts (Figure 1), indicating a severe malfunction of the official reporting system. Our study also found a considerable incompleteness in the health facility records. Of the newborns that died in a district hospital, 45% (27 of 60) were not noted as dead in the corresponding registers (Table 2). The corresponding figure for third level hospitals (PH and UBGH) was 15%. There seems to be many reasons for this under-reporting of neonatal deaths. One reason noted was the structure of the reporting system, where the responsibility for reporting was shifted away from the health system towards the parents of the newborn. There also seemed to be a poor understanding of the reasons and benefits of reliable statistics at all levels in the health system. In Table 3 we outline some of the reasons found during data collection.

**Table 2:** Neonatal deaths in Quang Ninh province, Vietnam, 2005, identified by different informants and methods of data collection according to place of death. Row percentages relating to the place of death are not cumulative, since neonatal deaths were identified from over-lapping sources.

**Place of Death**	**Neonatal deaths**	**Source of information**			
		
		**Hospital register**	**CHC register**	**CHC staff**	**VHWs**
**Regional and provincial hospital**	151	129 (85%)	57 (38%)	59 (39%)	94 (62%)
**District hospital**	60	33 (55%)	22 (37%)	32 (53%)	43 (72%)
**Community Health Center**	3	0 (0%)	3 (100%)	3 (100%)	2 (67%)
**Home or outside health facility**	67	3 (4%)	23 (34%)	31 (46%)	60 (90%)

**Total**	281*	168 (59%)	105 (37%)	126 (44%)	202 (71%)

*Information on place of death was missing in three cases

**Table 3:** Reasons found for under-reporting births and neonatal deaths in Quang Ninh province, Vietnam, 2005.

Local level
• Poor understanding of the rationale and importance for registering among health staff and families.
• Poor access to registrars.
• Difficulties in defining a neonatal death as opposed to a stillbirth.
• Local health staff having poor access to data due to family seclusion and high mobility in society.
• Reports by Village Health Workers being based on verbal reporting.

Health system level
• Reports being based on aggregates and not on individual data, making cross checking and additions impossible.
• The responsibility of reporting not being clearly communicated within the health system.
• Inadequate report forms.
• Infant mortality, but not neonatal mortality, being used in national statistics and surveys.
• Families and not the health system being ultimately responsible to register a birth or death.

## Discussion

We have found a substantial under-reporting of neonatal deaths in a province in Northern Vietnam. The NMR found through the different methods used in this study is four times higher than the officially reported NMR. The distribution of deaths over age is in accordance with international experience. The rule of two thirds apply to our data: that at least two thirds of neonatal deaths occurred in the first week of delivery [16] and that two thirds of the early neonatal deaths happened within the first 24 hours, a proportion that has increased over the past few years [2]. A tendency to systematically misreport the age of death, with so called "heaping" on day 7 has also been reported and used as a measure of the accuracy of collected data[17]. Our material contained no such heaping.

Nevertheless, our study has limitations, one being that we, in the pursuit of neonatal deaths, many times were depending on the malfunctioning reporting system we were trying to validate. Further, data collectors were dependent on the goodwill of local authorities to be able to get hold of medical records and to talk with health staff. The support from the Ministry of Health was very valuable in this regard. The VHWs were an important source for finding unreported neonatal deaths and the fact that 7% of them did not participate in group interviews implies that the results are not complete. There is also a question of recall bias, since the VHWs were asked to remember events more than a year old. This was dealt with by cross-checking and comparing with other sources whenever possible. However, correcting the possible errors of our study would only increase the NMR further.

The occurrence of this kind of under-reporting of neonatal deaths is commonly known, but the extent of the problem differs. Our results indicate that this is not an isolated problem and our findings verify earlier research on death reporting from various settings[13,18-24].

### Reasons for under-reporting

There seems to be various reasons why children are registered late or never (Table 3). In the Vietnamese society the responsibility to register newborns is put on the families, which in many cases have a poor understanding of the necessity to register. Many parents simply do not see an urgent need for the procedure and some in remote areas simply do not have easy access to registrars. Another important aspect, despite being specific to Vietnam, is that Vietnam's family planning law has implemented fines and other penalties for families having more than two children. Consequently, many families try to avoid extra payments and the third and following children go unreported [15]. According to a UNICEF study, less than half of the families had registered their child within the legally prescribed time period[15]. If instead the responsibility to report births and deaths was placed on the institutions where these events occurred, more reliable statistics would be produced. Our study shows that 225 out of the 284 neonatal deaths would have been reported if the deaths that were noted and known of at various institutions had been reported (including 60 neonatal deaths known by VHWs to have happened at home). This means that 79% instead of 25% of the neonatal deaths would have been included in the official statistics. Similar results have been reported elsewhere[18]. However, even if the hospitals were to report directly to the corresponding administrative body, their reports would be based on aggregates and not on individuals, leaving no mechanism for cross checking and adding omitted cases to the official statistics.

Another well-recognised difficulty in the registration of neonatal deaths is the matter of defining early neonatal deaths from stillbirths. To distinguish whether a baby dies intrapartum or shows signs of life right after delivery can be difficult, and establishing the boundary can be a source of confusion[15]. To avoid this, perinatal mortality has often been used as measure, combining the stillbirth rate with the early neonatal mortality rate. Especially in a setting with a large proportion of home deliveries, perinatal mortality has been a widely used indicator, avoiding the troubles of definition. However, considering the differences in etiology between stillbirths and neonatal deaths and the subsequent possible preventive interventions there is an argument for stratifying them[25].

The mobility in society also posed a problem for the VHWs and CHC staff to gather information. The antenatal care (ANC) in the province is not centralised or continuous and a pregnant woman can chose many different providers of ANC. The ANC journal also follows the woman and is not kept at a specific health facility. This, together with the habit of many pregnant Vietnamese women to go back to their parents' house in time for delivery, makes it difficult for VHWs and CHC staff to get an overview and follow-up on pregnancy outcomes.

### Consequences of under-reporting

Under-reporting of births and deaths may have severe consequences for policy formation, health planning, research and resource allocation at all levels in the pursuit of improved neonatal survival (Table 4). As in this example of Vietnam, the country does not qualify among the 60 priority countries set up by the Partnership for Maternal, Newborn and Child Health[26], thereby not receiving the attention given to these countries, despite being in a similar situation[1]. The priority list is based on WHO statistics [12] and with a more accurate reporting system, Vietnam, with its annual 1,6 million births, would be included among the countries in which more than 90% of all child deaths take place. Furthermore, monitoring of indicators for the Millennium Development Goal on child survival (MDG-4) will remain arbitrary as long as figures are based on estimations and extrapolations only and not on locally based reporting systems.

**Table 4:** Observed consequences of poor registration systems on neonatal health.

International and national levels
• The magnitude of neonatal mortality in the country is underestimated, and the flow of resources from major stake-holders is misguided
• The monitoring of indicators for the Millennium Development Goals will be arbitrary without valid information.
• Targeting of interventions to those most in need will be impossible.

Regional and local levels
• Awareness of neonatal mortality as a major health problem will be low, and interventions proved to reduce neonatal mortality will not be implemented.
• If any, measures taken to improve reproductive health will be inadequate since the interventions needed differ substantially between high and low mortality settings.
• At a local level, the perinatal period will not be perceived as a period of increased risk for mother and child, resulting in poor preparations and precautions for pregnancy and delivery.

At a national level, there seems to be a general understanding within the Vietnamese health system that the quality of data on births and deaths are poor. Even if there is a structure for a vital registration system in Vietnam, the authorities conduct an annual national survey on births and deaths but without collection of information and reporting on neonatal deaths[27]. The lack of accurate information on neonatal mortality results in a situation where health planners and decision-makers at national and regional levels cannot be guided or encouraged to act according to the real situation. Variations between different areas within regions might be very large, as we have found in Quang Ninh province, and without statistics based on the local situation it will be difficult to target interventions reach those in greatest need.

At a local level, the lack of reliable statistics will be a factor that preserves status quo. Pregnant women will not prepare for or take adequate precautions before delivery since pregnancy may be perceived as a part of life and not as a potentially hazardous situation[28]. Initiatives by local actors or NGOs will not be backed up by statistics and local authorities will not act to solve a problem they do not perceive they have. On the contrary, local authorities might even encourage under-reporting in order to gain approval and rewards from higher levels.

To register the death of a newborn is, however, not only a matter of statistics, but also a matter of human rights. The right to birth registration is part of the UN convention on the Rights of the Child [29] but despite this fact, newborns that are not reported to have died are often not registered as being born. The implementation and enforcement of human rights depends on civil registration, something that in many parts of the world is not well known or recognized[30]. Acknowledging and reporting a neonatal death strengthens the position and rights of the living by acknowledging that every child is a human being from the moment of birth. The UN Millennium Project states that in order to improve the situation of women's and children's health in the world, human rights need to be highlighted and enforced[31]. Pursuing a true representation of child survival is an integral part of this effort[32].

## Conclusion

In order to reach MDG-4 and improve child survival, neonatal mortality must be targeted. One part of that effort is to improve and build trustworthy reporting systems for births and deaths in countries with incomplete official data. Meanwhile, it is recommended that planners and politicians at all levels take the under-reporting into account in order to avoid basing decisions on invalid information. In doing so, the challenges in neonatal health can be recognised and resources may be distributed according to real needs instead of overly optimistic indicators.

## Competing interests

The author(s) declare that they have no competing interests.

## Authors' contributions

All authors were involved in the conception and design of the study. NTN, DPH and LE organised and supervised data collection. MM drafted the paper with contributions from the co-authors. All authors read and approved the final manuscript.

## Pre-publication history

The pre-publication history for this paper can be accessed here:


